# Supplementary figures and images for: Effects of sea ice and wind speed on phytoplankton spring bloom in central and southern Baltic Sea
Source: PLoS One. 2021 Mar 3;16(3):e0242637. doi: 10.1371/journal.pone.0242637 (PMC7928518; doi:10.1371/journal.pone.0242637)

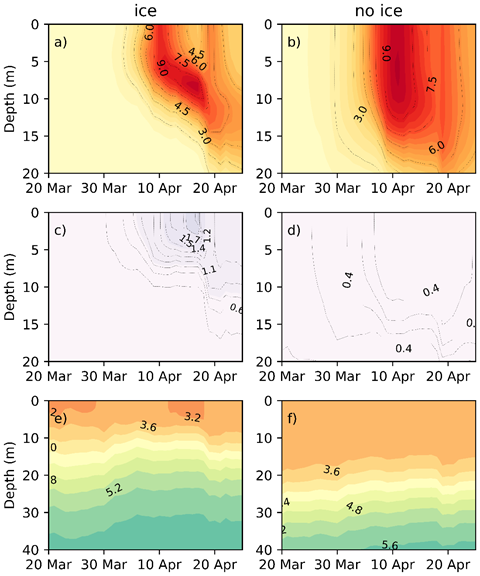

Supplement: S1 Fig — (a) Diatoms (no ice run); (b) diatoms (ice run); (c) dinoflagellates (no ice run); (d) dinoflagellates (ice run); (e) and (f) are water densities (kg/m3) with ice run and no ice run. The water density is defined as the sigma density [(density(t,s,z)– 1000)] kg/m3. (TIFF) [file pone.0242637.s001.tiff]

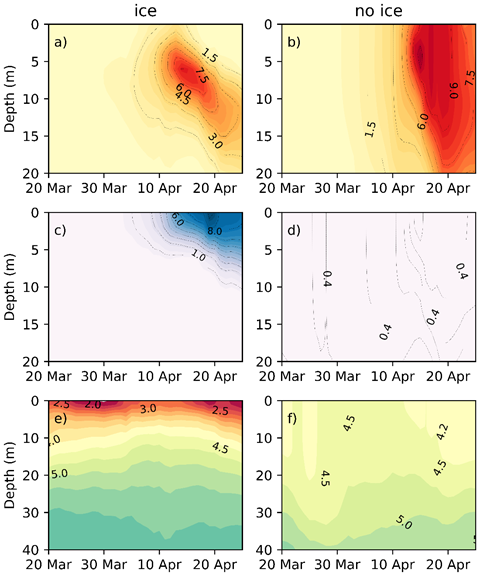

Supplement: S2 Fig — (a) Diatoms (no ice run); (b) diatoms (ice run); (c) dinoflagellates (no ice run); (d) dinoflagellates (ice run); (e) and (f) are water densities (kg/m3) with ice run and no ice run. The water density is defined as the sigma density [(density(t,s,z)– 1000)] kg/m3. (TIFF) [file pone.0242637.s002.tiff]

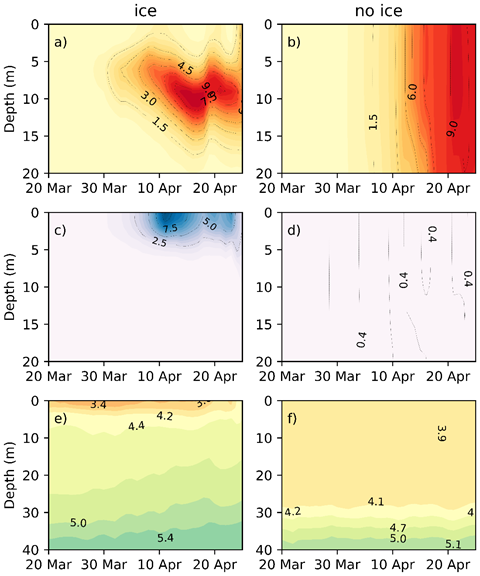

Supplement: S3 Fig — (a) Diatoms (no ice run); (b) diatoms (ice run); (c) dinoflagellates (no ice run); (d) dinoflagellates (ice run); (e) and (f) are water densities (kg/m3) with ice run and no ice run. The water density is defined as the sigma density [(density(t,s,z)– 1000)] kg/m3. (TIFF) [file pone.0242637.s003.tiff]

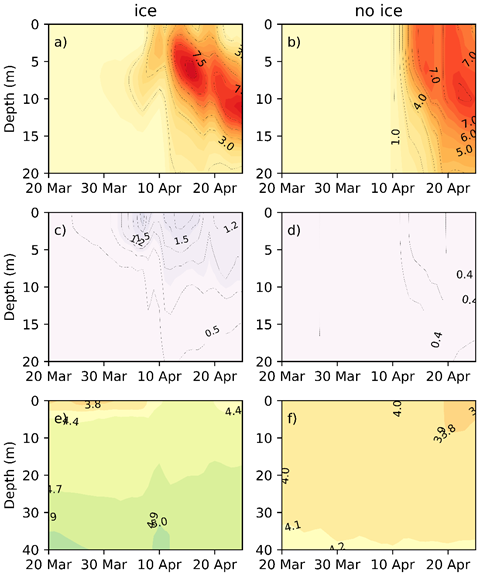

Supplement: S4 Fig — (a) Diatoms (no ice run); (b) diatoms (ice run); (c) dinoflagellates (no ice run); (d) dinoflagellates (ice run); (e) and (f) are water densities (kg/m3) with ice run and no ice run. The water density is defined as the sigma density [(density(t,s,z)– 1000)] kg/m3. (TIFF) [file pone.0242637.s004.tiff]

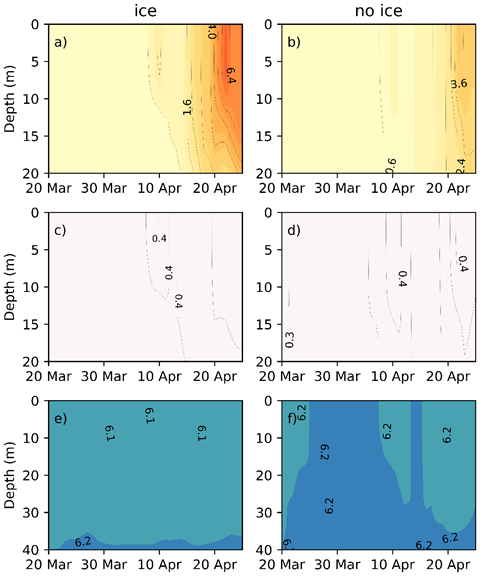

Supplement: S5 Fig — (a) Diatoms (no ice run); (b) diatoms (ice run); (c) dinoflagellates (no ice run); (d) dinoflagellates (ice run); (e) and (f) are water densities with ice run and no ice run. The water density is defined as the sigma density [(density(t,s,z)– 1000)] kg/m3. (TIFF) [file pone.0242637.s005.tiff]

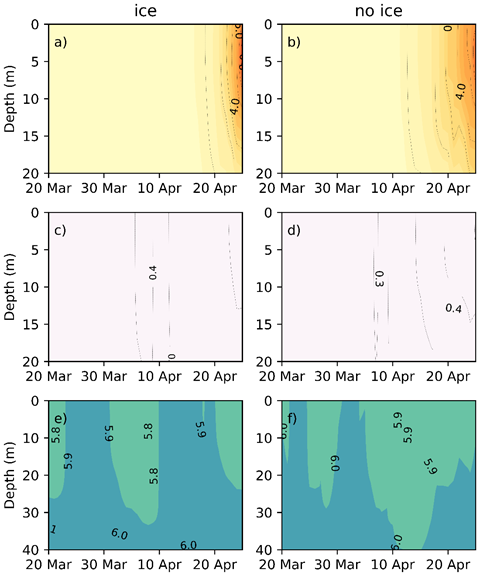

Supplement: S6 Fig — (a) Diatoms (no ice run); (b) diatoms (ice run); (c) dinoflagellates (no ice run); (d) dinoflagellates (ice run); (e) and (f) are water densities with ice run and no ice run. The water density is defined as the sigma density [(density(t,s,z)– 1000)] kg/m3. (TIFF) [file pone.0242637.s006.tiff]

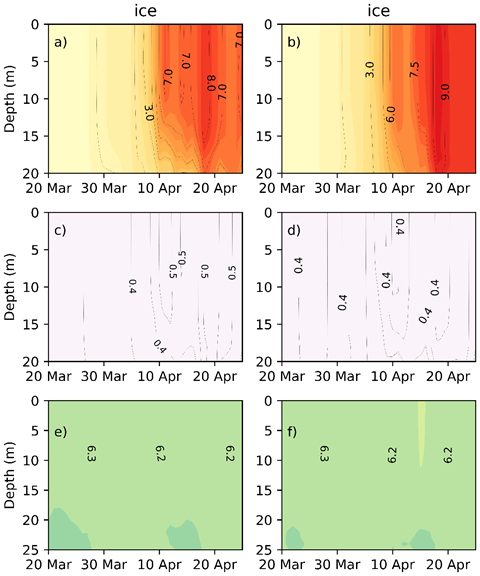

Supplement: S7 Fig — (a) Diatoms (no ice run); (b) diatoms (ice run); (c) dinoflagellates (no ice run); (d) dinoflagellates (ice run); (e) and (f) are water densities with ice run and no ice run. The water density is defined as the sigma density [(density(t,s,z)– 1000)] kg/m3. (TIFF) [file pone.0242637.s007.tiff]

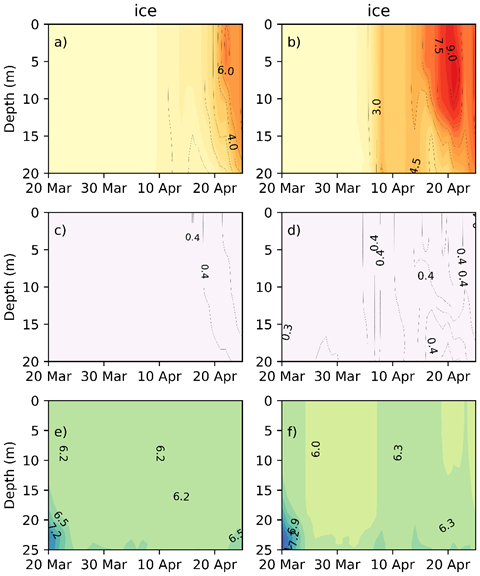

Supplement: S8 Fig — (a) Diatoms (no ice run); (b) diatoms (ice run); (c) dinoflagellates (no ice run); (d) dinoflagellates (ice run); (e) and (f) are water densities with ice run and no ice run. The water density is defined as the sigma density [(density(t,s,z)– 1000)] kg/m3. (TIFF) [file pone.0242637.s008.tiff]
