# Supplementary material for: Effects of sea ice and wind speed on phytoplankton spring bloom in central and southern Baltic Sea
Source: PLoS One. 2021 Mar 3;16(3):e0242637. doi: 10.1371/journal.pone.0242637 (PMC7928518; doi:10.1371/journal.pone.0242637)
Supplement: S1 Table — Data from Baltic Nest, ICES and satellite data from Copernicus database. (DOCX) [file pone.0242637.s009.docx]

**S1 Table. Peak of chlorophyll-a in April 2013.**

| **Station** | **Model** | **Sat** | **ICES or Baltic Nest** |
| --- | --- | --- | --- |
| **14** | 8.6 | 2.4 | 17 |
| **H1** | 9.4 | 2.9 | 7.9 |
| **32** | 8.1 | 7.5 | 3.9 |
| **G1** | 10.4 | 5.8 | 15 |
| **OMBPK3** | 5.9 | 4.3 | 7.2 |

Data from Baltic Nest, ICES and satellite data from Copernicus database.
