# Supplementary material for: Effects of sea ice and wind speed on phytoplankton spring bloom in central and southern Baltic Sea
Source: PLoS One. 2021 Mar 3;16(3):e0242637. doi: 10.1371/journal.pone.0242637 (PMC7928518; doi:10.1371/journal.pone.0242637)
Supplement: S2 Table — Phytoplankton events based on observations. Cases, which developed under ice cover, as illustrated in figures, have been marked with an asterisk. Stations location A (58.35°N;18.14°E) and B (57.3°N;20.1°E). Ice concentration for up to 10 days before measurements. (DOCX) [file pone.0242637.s010.docx]

**S2 Table. The table shows dia/dino index and records stating the existence of ice.**

| **Time** | **Index** | **Station** | **Ice cover (%)** |
| --- | --- | --- | --- |
| **1990 March 7 and 27** | 0.57 | A | 0 |
| **1991 March 12 and 26** | 0.45 | A | 0 |
| **1992 March 17** | 0.56 | A | 0 |
| **1994 March 16 and 28** | 0.55 | A | 0-100 |
| **1995 March 15 and 20** | 0.5 | A | 0 |
| **1996, February 24** | 0.003^*^ | B | 5-90 |
| **1996, March 12** | 0.35^*^ | A | 0-30, on the border of the fast ice |
| **1997 March 18** | 0.56 | A | 0 |
| **1998 March 9 and 24** | 0.2 | A | 0 |
| **1999 March 2 and 16** | 0.52 | A | 0 |
| **2000 March 14 and 28** | 0.5 | A | 0 |
| **2001 March 3 and 14** | 0.4 | A | 0-30, on the border of the fast ice |
| **2002 March 7 and 26** | 0.51 | A | 0 |
| **2003 February 20** | 0.3^*^ | B | New ice (up tu10 cm) |
| **2003 March 11 and 22** | 0.02^*^ | A | 5-30 |
| **2004 February 20** | 0.25^*^ | B | New ice |
| **2004 March 10** | 0.3^*^ | A | New ice |
| **2005 March 14 and 30** | 0.45 | A | 0 |
| **2006 March 4 and 29** | 0.52 | A | 0 |
| **2007 March 14 and 28** | 0.55 | A | 0 |
| **2008 March 12 and 27** | 0.52 | A | 0 |
| **2009 March 11 and 25** | 0.4 | A | 0 |
| **2010 March 17** | 0.45^*^ | A | 5-50 |
| **2011 March 11** | 0.34^*^ | A | 5-50 |
| **2012 March 3 and 14** | 0.5 | A | 0 |
| **2013 March 11** | 0.13^*^ | A | 0-70, New ice |
| **2014 March 12 and 27** | 0.51 | A | 0 |
| **2015 March 9, 23, 30** | 0.4 | A | 0 |
| **2016 March 15 and 30** | 0.51 | A | 0 |
| **2017 March 13, 21, 27** | 0.5 | A | 0 |
| **2018 March 12, 19, 26** | 0.51 | A | 0 |
| **2019 March 12 and 28** | 0.55 | A | 0 |

Phytoplankton events based on observations. Cases, which developed under ice cover, as illustrated in figures, have been marked with an asterisk. Stations location A (58.35°N;18.14°E) and B (57.3°N;20.1°E). Ice concentration for up to 10 days before measurements.
