# Supplementary material for: Effects of sea ice and wind speed on phytoplankton spring bloom in central and southern Baltic Sea
Source: PLoS One. 2021 Mar 3;16(3):e0242637. doi: 10.1371/journal.pone.0242637 (PMC7928518; doi:10.1371/journal.pone.0242637)
Supplement: Attachment — Submitted filename: Response to Reviewers.docx [file pone.0242637.s011.docx]

Response to Reviewers

Our sincere thanks for the relevant suggestions and comments that improve the quality of the manuscript and open up new perspectives.

***1.1 Reviewer #1:*** *These conclusions are certainly interesting and even intriguing.*  *The explanations given by the authors make sense but it remains puzzling for the world ocean where many areas never experience ice but have with huge dinoflagellate blooms and, on the contrary, areas of the Arctic and Antarctic oceans with ice are often dominated by diatoms. It is difficult to understand why the Baltic Sea would be such a peculiar case.*

The focus of our manuscript is indeed on the influence of ice and wind. However, the mechanism of dominance of diatoms or dinoflagellates depends on many other factors such as water density (salinity, temperature), circulation characteristics (like upwelling), wind (water turbulent mixing), nutrient concentrations and balance, as well as phytoplankton physiology.

Example, 3-4 days of wind silence (wind speed <5 m / s) can be very favorable for dinoflagellates.

We strongly agree that there are open points for discussion and further research (specifically multi-year and climate scenario simulations) and ecosystem model development are needed for parametrization of phytoplankton.

***1.2 Reviewer #1:*** *“ However, it is quite possible that the inherently crude parametrization of the dynamics of diatom and dinoflagellate populations leads to these model results. Natural diatom and dinoflagellate populations have huge variations in their characteristics, e.g. cell size, growth and sinking speed, vertical mobility, etc. and parameters fixed to some estimates may not lead to realistic outcomes of the model.”*

We fully agree with the reviewer that there are huge variations in the phytoplankton community. However, the model is inevitably a highly simplified representation of reality, where dominant types of phytoplankton are grouped according to similar characteristics (ref ERGOM papers). Updating the model according to up-to-date knowledge on phytoplankton ecology and physiology (including production of cysts), and possibly diversifying functional types of phytoplankton to include higher variability, are necessary to improve its performance in the future.

***1.3 Reviewer #1:*** *The manuscript is definitely too long and looks more like a report.*

We would like to thank the reviewer for his comment and pointing this out.

The paragraph “**Ice Conditions during Spring Bloom**” has now been removed from the paper to shorten its overall length.

We have reformulated the discussions and have specified the scope of the paper.

***2.1 Reviewer #2:*** *Getting the model to work with the slower growing dinoflagellates can be difficult, and it seems they had to increase the growth rate above what is published under optimal conditions for dinoflagellates.*

*L123. 0.7 d-1 is likely too high, 0.4 d-1 is more realistic*

We agree with the reviewer that the values of the phytoplankton parameters need further improvement and the development of the model is in progress.

When we used 0.4 d-1 in the model, the dia/dino index in the results obtained was not a close match with the results of Wasmund [1]. However, after changing the value to 0.7 d-1, we were able to obtain results which were a closer match to the results of Wasmund.

Therefore, we have used the value of the Ecological Regional Ocean Model (IOW ergom) 0.7 [2] which is used by the The Leibniz Institute for Baltic Sea Research, Warnemünde.

***2.2 Reviewer #2:*** *“Also, large parts of the central Baltic Sea is very often without ice and still dinoflagellates seem to dominate, see e.g. Fig 4 in Klais et al 2011. So the distribution of dinoflagellate dominance extends from typically ice covered to not ice covered.”*

Diatoms need less light and have a higher growth rate in the model, so they are more dominant. According to the model, dinoflagellates can only grow if the concentration of diatoms is low enough. This allows more light on the sea surface leading to the dominance of dinoflagellates. This is a situation that can exist in the central parts of the Baltic Sea.

Another situation that can occur is when diatoms sink deep from the surface. Such conditions occur when there is ice in the area or when the daily average wind speed is less than 5 m/s. The circulation is also important.

In view of the foregoing, despite the central Baltic Sea often being without ice, dinoflagellates still seem to dominate because of the low wind speed that is not fully mixing the sea surface layers and ensuring that diatoms sink to the bottom. Also, the concentration of the diatoms is principally smaller (compared to the coastal areas) allowing the dinoflagellates to absorb light creating optimal conditions for their growth and dominance.

***2.3 Reviewer #2:*** *“L483-486: There is ample evidence dinoflagellates dominate even under ice free winters so I do not buy this conclusion, a more interesting question is why the model gets it wrong.”*

The reviewer is correct that the algal spring bloom in the Baltic Sea was often characterised by the dominance of dinoflagellates over diatoms. However, in our experiments, in ice free open sea areas, often diatoms were found to dominate. On the other hand, the stratification caused in the water due to several variables would lead to a situation favouring the dominance of dinoflagellates, but this is potentially more important for coastal regions.

We understand that the model is not perfect but it has been reliable in the experiments we have conducted so far. However, this paper would give impetus to further the development of the model.

***2.4 Reviewer #2:*** *“What I think is something missing in the model is the fact that the spring bloom is driven by the inflow of freshwater creating the initial stratification rather than thermal stratification which requires longer time for the surface layer to warm up.”*

Yes, freshwater runoff has importance for the spring bloom. Inflow of freshwater is considered in the model and it plays a role with regard to nutrient input and stratification. However, in our modelling experiment we did not analyse the effects of freshwater inputs separately, focusing on integrated change in the upper water column dynamics due to lack of ice. As we were only simulating a single spring bloom event, the effect of freshwater inputs might have been not pronounced, and would be more apparent in long-term simulations, which we are currently considering as a follow-up to this work.

***2.5 Reviewer #2:*** *“L84-97: The difference in growth rate is the same between dia and dinos in the Baltic Sea (Spilling and Markager 2008 - https://doi.org/10.1016/j.jmarsys.2006.10.012).*

Many thanks for the relevant article references.

In the model we have used different growth rates for diatom and dinoflagellate, 0.7 d-1 and 1.3 d-1 respectively. We have increased the growth rate of dinoflagellate from 0.4 to 0.7 [2, Neumann 2002]. According to the reference, it seems that a reduction in the growth rate of the diatom would have been more justified.

***2.6 Reviewer #2:*** *What is likely a factor is timing of requirement (Kremp et al 2008) and stratification. What is different in the Baltic Sea from many other coastal regions is the massive freshwater outflow at spring and salinity stratification rather than thermal stratification playing an effect (Stipa 2002 & 2004) which is important for the start of the spring bloom.*

Our model does take into account the outflow of freshwater. However, our modelling experiment with and without ice cover demonstrates that presence/absence of ice as an important factor shaping the composition of phytoplankton spring bloom. We certainly agree that there are other factors at play which will influence these dynamics in the future, including changes in atmospheric forcing, runoff and nutrient balance. We have changed relevant parts of the text to clarify this.

***2.7 Reviewer #2:*** *“L 75: Could add a line explaining that diatoms are non-motile and sink quicker whereas dinoflagellates are motile and can position themselves vertically.”*

Thank you, we changed the sentence in Introduction:

Wind is one of the factors governing vertical mixing within the euphotic layer ( ~9.6 m in Baltic, [29]) and in stagnant water, the diatoms, being non-motile and heavier, sink quickly, whereas the dinoflagellates are motile and can therefore position themselves vertically.

***2.8 Reviewer #2:*** L313: In reality dinoflagellates are actually the dominating group at least in station 32.

Phytoplankton composition has a great deal of variability, both in space and time.

For example, on 14 and 15 March 2002, 14 measurements were made in a small sea area (data from Irina Olenina), 20⁰5’-21⁰01’; 55⁰39’-56⁰01’ (not too far from station 32).

The mean biomass of the diatoms was 1024µG/L and the standard deviation was 880µG/L. The maximum value was 3425µG/L and the minimum was 71µG/L. The mean value of the dinoflagellates was 38µG/L.

However, only a month later, the mean value of the dinoflagellates was 972µG/L, (std= 915µG/L, min=176, max = 2710), while the mean value of the diatoms was 420µG/L (std=519, min 24, max=1308).

Therefore, it can be seen that one station cannot be fairly representative of which phytoplankton group is dominant during bloom when the measurements are taken only 1 or 2 times during spring.

***2.9 Reviewer #2:*** L440-41: no they actually swim

Agreed; however, for simplicity, the model formulation does not take the ability of dinoflagellates to swim into account. We believe this assumption is justified given their slow speed of movement and the spatial resolution of the model applied in this study.

***2.10 Reviewer #2:*** L477-479: sounds like there was no spring bloom in the past

We agree that the sentence was not clearly formulated. We have changed this sentence:

1. In the past, at the beginning of spring, the sea ice was so thick that the lack of light limited the growth of the phytoplankton [50]. Therefore, phytoplankton spring blooms occurred later, in the middle or end of May. Diatoms dominated in the southern part of the Baltic Sea where sea ice was absent.

References

1. Wasmund N. The diatom/dinoflagellate index as an indicator of ecosystem changes in the baltic sea. 2. historical data for use in determination of good environmental status. Frontiers in Marine Science. 2017 Jun 8;4:153.
2. Neumann T, Fennel W, Kremp C. Experimental simulations with an ecosystem model of the Baltic Sea: a nutrient load reduction experiment. Global biogeochemical cycles. 2002 Sep;16(3):7-1.

------------------------------------------------------------------------------------------

**Discussion**

Sea ice eliminates the effect of wind on water circulation, thus, affecting water mixing and currents; air does not come in contact with the water surface and sunlight is also reflected back into the atmosphere. We compared the results of numerical experiments performed to study the effect of the absence of ice on the water body in freezing temperatures and the presence of sea ice to assess the impact of sea ice. The results showed that the spring bloom of dinoflagellates appeared only in the sea area with thin ice (or low wind conditions) while diatoms dominated in ice-free water.

Phytoplankton distribution is determined by the availability of nutrients, light, and temperature for growth, predation, sedimentation rate, and migration [43], as well as prevailing hydrodynamic conditions. The studied hydrodynamic conditions caused by wind speed, such as stratification, water mixing and currents, played a crucial role in the formation and location of phytoplankton biomass. In the modelling experiments, seasonal sea ice cover reduced wind-driven mixing and allowed the development of under-ice stratification (Figs 6 and 9). When water mixing is weak, large diatom cells sink while dinoflagellate cells remain in the surface layers because they exhibit neutral buoyancy. It may be noted that light can also be a limiting factor for dinoflagellates in the presence of diatoms, favouring the dominance of the latter.

In the present model nutrients most likely did not play a role because all simulations had the same nutrient input and initial conditions. However, the dynamics of DIN and DIP (Descriptor 5 of MSFD) were different for diatoms and dinoflagellates. When the predominant diatoms with a higher growth rate quickly consumed nutrients, it indicated the end of the spring bloom. When physical conditions for dinoflagellates were suitable, their nutrient intake was lower than diatoms and therefore the nutrients were available for longer in the euphotic zone. Diatoms lost their competitive advantage under sea ice and calm wind, as these conditions favoured sinking into the deeper layers of water where light was not available. Accordingly, the model results demonstrate how the changes in the sea ice or wind conditions drive changes in the Dia/Dino index.

The changes in dominance of these two phytoplankton classes affects the marine food web (Descriptor 4 of MSFD), and a low Dia/Dino index has been assumed by HELCOM to be an indicator of eutrophication (Descriptor 5 of MSFD) [2]. However, the results of the conducted modelling experiment show that during spring bloom low dia/dino index can be strongly influenced by physical factors and therefore should not be considered an indicator of eutrophication.

Potential reasons for dinoflagellate-dominated spring bloom becoming more frequent in the last decades can be summarised as follows:

1. Relatively thin ice: Studies have reported that ice conditions have gradually become milder over the years. However, the monitored observations for ice thickness around the Baltic Sea coast reveal both decreasing and increasing trends [13]. In the Gulf of Finland, a thinning trend of -25 cm has been observed [44]. Sea ice in the middle of the Baltic Sea plays a crucial role in dinoflagellate-dominated spring bloom. A similar result was found by Klais et al. [20] which indicated that the local physical conditions (mild and variable winters) resulting from large-scale changes in weather patterns may be more crucial factors than nutrients in explaining the shift towards dinoflagellate dominance.

2. Changeable weather in winter and spring: Since the 1990s, stratification collapse (strong mixing events) has frequently occurred in winters from October to April when saline and thermal stratification decreases [45]. Winters have become increasingly inconstant, with extremely high air temperatures for short periods [46]. High air temperatures cause sea ice to melt and reduce its thickness. When the temperature decreases again, the ice cover expands. The formation and closure of ice-free openings is a common phenomena in the Gulf of Finland [47-49].

Taking into account the results of this modelling experiment, the past, present, and the future of spring bloom in response to the reduction of ice cover due to climate change can be classified as follows:

1. In the past, at the beginning of spring, the sea ice was so thick that the lack of light limited the growth of the phytoplankton below it [50]. Therefore, phytoplankton spring blooms occurred later, typically in the middle or end of May. Diatoms dominated at the south of Baltic Sea where sea ice was absent.

2. By the end of the 1980s, the light conditions in the central Baltic Sea improved due to reduced ice thickness, and both diatoms and dinoflagellates began to grow. Dinoflagellates gained a competitive advantage here because diatoms, being heavier, sank rapidly.

3. According to future climate scenarios [14], the ice extent will be further reduced with shorter ice seasons and thinner ice cover. Completely ice-free winters are, however, unlikely to occur during the 21st century [51]. Most recent studies predict that dinoflagellates will dominate the spring bloom in the future. The modelling experiments conducted within this study, which only considered the changes in ice cover, demonstrated that loss of ice can be a factor promoting diatom dominance during spring bloom via changing the mixing regime.

Our modelling experiments were conducted over one growing season only. Longer-term model runs are necessary to elucidate whether the observed shifts towards diatom dominance during spring bloom are the initial reaction of the model to experimental perturbations, which will be negated by shifts in large-scale circulation and stratification caused by loss of sea ice, as well as alterations in nutrient availability and balance.

Moreover, rather simplistic parameterisation of phytoplankton groups in the model applied is a limiting factor preventing more comprehensive answers regarding the future of phytoplankton spring blooms. To address this, in the future studies model formulation should be updated with the up-to-date understanding of the Baltic Sea phytoplankton ecology, especially formation of resting cysts, as well as consider wider diversity of phytoplankton functional groups.

**Conclusions**

According to the study, ice conditions preceding the spring bloom influence the species composition of phytoplankton during spring bloom. In the presence of ice cover, which eliminates the effect of wind in the upper layer of water, the heavier particles (diatoms) sink below the euphotic zone (~ 10m) favouring proliferation of dinoflagellates. However, if diatoms remain in the upper layer of water due to turbulent mixing induced by wind, they dominate the phytoplankton spring bloom.

The changing ice conditions are only one of the key factors directly affecting timing and composition of phytoplankton spring bloom within the Baltic Sea with cascading consequences for nutrient transfer and the entailing ecological dynamics.

The modelling experiments have demonstrated that the dia/dino index in spring is strongly influenced by physical factors, therefore further research is required to confirm the robustness of this index as an indicator of eutrophication[4,8]. Since conclusions of this work are based on short-term model simulations, it should be regarded as an impetus for the future phytoplankton modelling studies that should test the long-term consequences of ice loss for phytoplankton bloom dynamics in the Baltic Sea using forecast scenario simulations.
